# miR-4721, Induced by EBV-miR-BART22, Targets *GSK3β* to Enhance the Tumorigenic Capacity of NPC through the *WNT/β-catenin* Pathway

**DOI:** 10.1016/j.omtn.2020.09.021

**Published:** 2020-09-23

**Authors:** ZiBo Tang, WeiFeng Chen, Yan Xu, Xian Lin, Xiong Liu, YongHao Li, YiYi Liu, ZhiJian Luo, Zhen Liu, WeiYi Fang, MengYang Zhao

**Affiliations:** 1 Cancer Center, Integrated Hospital of Traditional Chinese Medicine, Southern Medical University, 510315 Guangzhou, China; 2 Department of Otolaryngology, Head and Neck Surgery, Nanfang Hospital, Southern Medical University, 510515 Guangzhou, China; 3 Key Laboratory of Protein Modification and Degradation, School of Basic Medical Sciences, Affiliated Cancer Hospital and Institute of Guangzhou Medical University, 511436 Guangzhou, China; 4 Department of Oncology, The People’s Hospital of Zhengzhou University, 450003 Zhengzhou, China

**Keywords:** epithelial cell carcinoma, EBV, onco-miR, cell cycle, proliferation, tumor marker, GSK3β, *WNT/β-catenin* Pathway

## Abstract

Nasopharyngeal carcinoma (NPC) is prevalent in East and Southeast Asia. In a previous study, Epstein-Barr virus (EBV)-miR-BART22 induces tumor metastasis and stemness and is significantly involved in NPC progression. In the present study, we observed that miR-4721 is induced by EBV-miR-BART22 through phosphatidylinositol 3-kinase (PI3K)/AKT/c-JUN/Sp1 signaling to promote its transcription. In a subsequent study, we observed that miR-4721 serves as a potential oncogenic factor promoting NPC cell cycle progression and cell proliferation *in vitro* and *in vivo*. Mechanism analysis indicated that miR-4721 directly targetes GSK3β and reduces its expression, which therefore elevates β-catenin intra-nuclear aggregation and activates its downstream cell cycle factors, including CCND1 and c-MYC. In clinical samples, miR-4721 and GSK3β are respectively observed to be upregulated and downregulated in NPC progression. Elevated expression of miR-4721 is positively associated with clinical progression and poor prognosis. Our study first demonstrated that miR-4721 as an oncogene is induced by EBV-miR-BART22 via modulating PI3K/AKT/c-JUN/Sp1 signaling to target GSK3β, which thus activates the WNT/β-catenin-stimulated cell cycle signal and enhances the tumorigenic capacity in NPC. miR-4721 may be a potential biomarker or therapeutic target in NPC treatment in the future.

## Graphical Abstract

**Figure:**
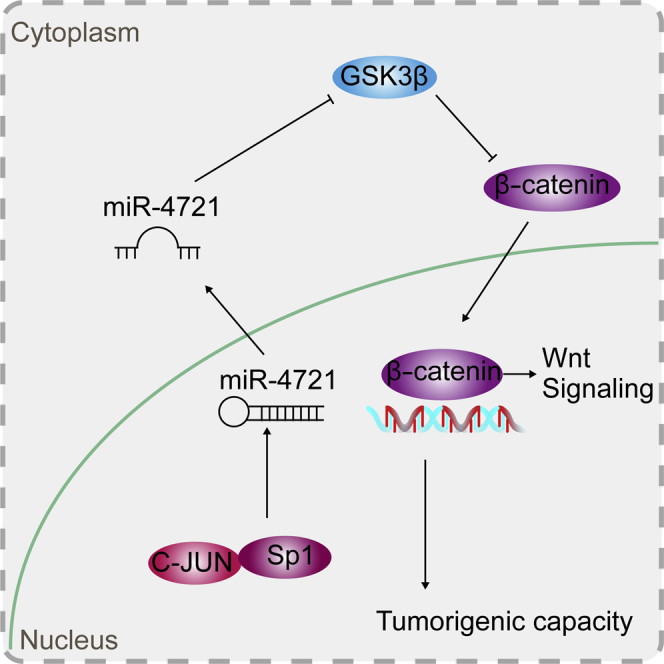

## Teaser

MicroRNAs play an important role in the tumorigenesis of nasopharyngeal carcinoma (NPC), with the regulations between two miRNAs being less reported. Tang and colleagues demonstrated that miR-4721, induced by EBV-miR-BART22, facilitates the progression of NPC. Further experiments both *in vitro* and *in vivo* clarified the underlying mechanism.

## Introduction

MicroRNAs (miRNAs) are a class of genes that are very conservative in evolution and wildly distributed across the genome. It is reported that these small non-coding RNAs are widely involved in development, immunity, metabolism, and especially cancers. In “Hallmarks of cancer: the next generation,” Hanahan and Weinberg^1^ summarized 10 characteristics of cancers from self-sufficiency in growth signals to genome instability and mutation, with miRNAs being involved in the regulation of all 10 hallmarks. Abnormal expression of miRNAs has been documented to be associated with inflammation and tumor progression.^2^, ^3^, ^4^, ^5^, ^6^, ^7^, ^8^, ^9^, ^10^ miR-4721 was first identified in 2011,^11^ yet knowledge about the regulatory function and its role in carcinogenesis is still lacking.

Nasopharyngeal carcinoma (NPC), which arises from the nasopharyngeal mucosal lining, is one of the epithelial cell carcinomas in head and neck cancer. According to the International Agency for Research on Cancer, more than 70% of NPC patients were diagnosed as having locoregionally advanced disease with an unfavorable prognosis,^12^ and thus it is indispensable to find a tumor marker for early detection for NPC patients. As an endemic disease prevalent in East and Southeast Asia,^13^^,^^14^ the non-keratinizing subtype of NPC comprises >95% of all such cases in the region, which are invariably related to Epstein-Barr virus (EBV) infection.^15^ Numbers of studies have shown that NPC is closely associated with EBV infection.^16^, ^17^, ^18^, ^19^ It was found that EBV-coded miRNAs, such as EBV-miR-BART1,^20^ BART2-5p,^21^ BART7,^22^ and BART13,^23^ are involved in tumorigenesis and NPC progression, and could be used as biomarkers or prognostic indicators. In a prior study, we found that EBV-miR-BART22 has a high expression level in NPC. It promotes tumor stemness and metastasis and was also found to be involved in cisplatin resistance by regulating the phosphatidylinositol 3-kinase (*PI3K*)/*AKT*/*c-JUN* pathway.^24^

In this study, we interestingly observed that miR-4721 is induced by EBV-miR-BART22. Further experiments explored the regulatory relationships of miR-4721 and confirmed its role in the carcinogenesis of NPC. Our study shows that miR-4721, as an oncogenic (onco-)miRNA, is involved in the EBV-miR-BART22 regulatory mechanism through a PI3K/AKT/c-JUN/Sp1 signaling axis to enhance the tumorigenic capacity by activating the *WNT*/*β-catenin* pathway. This newly identified miRNA might serve as a prognostic indicator in NPC treatment.

## Results

### miR-4721 Is Highly Expressed and Is Induced by EBV-miR-BART22 in NPC

**Figure 1:**
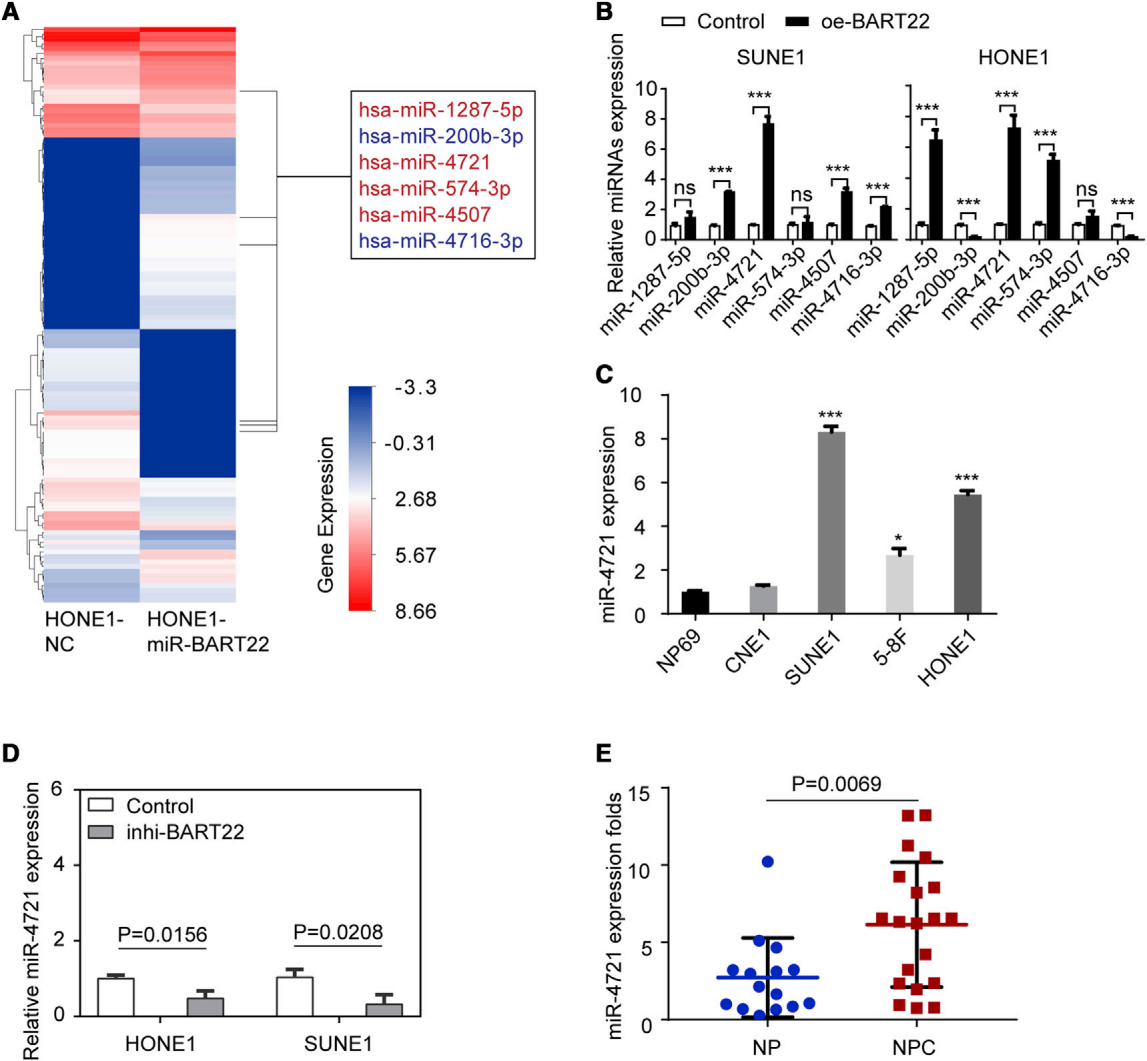
miR-4721 Is Highly Expressed in NPC and Is Induced by EBV-miR-BART22 (A) miRNA expression profile microarray screening. A supervised hierarchical cluster analysis of 120 differentially expressed miRNAs between HONE1-NC and HONE1-miR-BART22 is shown. There are 63 upregulated miRNAs and 57 downregulated miRNAs in HONE1-miR-BART22 compared to HONE1-NC. Left: heatmap of the 120 differentially expressed miRNAs. Red represents upregulated miRNAs and blue represents downregulated miRNAs. Right: six tops differentially expressed miRNAs. (B) Each miRNA’s relative expression normalized to U6 was detected by qRT-PCR in two NPC cells with/without EBV-miR-BART22 overexpression. The data are shown as the mean ± SD. ∗∗∗p < 0.001. ns, not significant. (C) The expression fold of miR-4721 in NP69 cells and in four NPC cell lines (CNE1, SUNE1, 5-8F, and HONE1). The data are shown as the mean ± SD. ∗p < 0.05, ∗∗∗p < 0.001. ns, not significant. (D) The relative expression (fold) of miR-4721 in inhibitor (inhi)-BART22 and negative control. The data are shown as the mean ± SD. ∗p < 0.05. (E) The expression of miR-4721 was higher in NPC tissues (n = 20) than in NP tissues (n = 15). Student’s t test. Mean ± SD.

EBV-miR-BART22 was reported to promote *PI3K*-*AKT* signaling pathway activation and NPC cell migration, invasion, stemness, and chemo-resistance in previous research.^24^ The fact that EBV-miR-BART22 drives NPC tumorigenesis calls for a better understanding at a different level. Thus, we conducted an Agilent human miRNA (8×60K, design ID: 070156) microarray chip analysis (Figure 1A) and found 63 upregulated miRNAs and 57 downregulated miRNAs, when compared to normal control (NC) samples. To verify the results of the miRNA chip, we re-examined the expression of six top differentially expressed miRNAs in HONE1-miR-BART22 and HONE1-NC cells (Figure 1B). Bioinformatics analysis of these six miRNAs showed diverse results. Based on the expression difference and survival analyses, miR-4721 was selected to be the candidate miRNA in our further study. We then detected miR-4721 expression in four NPC cell lines (CNE1, SUNE1, 5-8F, HONE1) and healthy NP cell lines (NP69) by qRT-PCR. It appears that miR-4721 is highly expressed in NPC cells (Figure 1C).

To further validate the relationship between miR-4721 and EBV-miR-BART22, we decreased EBV-miR-BART22 expression using BART22-inhibitor in two NPC cell lines and found that miR-4721 expression declined in both (Figure 1D).

To detect miR-4721 expression level in human tissues, we collected 15 normal nasopharyngeal epithelial tissues and 20 NPC tissues, and we found that miR-4721 expression is elevated in NPC tissues compared with healthy NP tissues (p = 0.0069; Figure 1E).

These results suggest that miR-4721 is highly expressed in NPC and that its expression is induced by EBV-miR-BART22 in this regulation process.

### miR-4721 Promotes NPC Proliferation *In Vitro* and *In Vivo*, and the miR-4721 Inhibitor Induced G_1_ Phase Arrest

**Figure 2:**
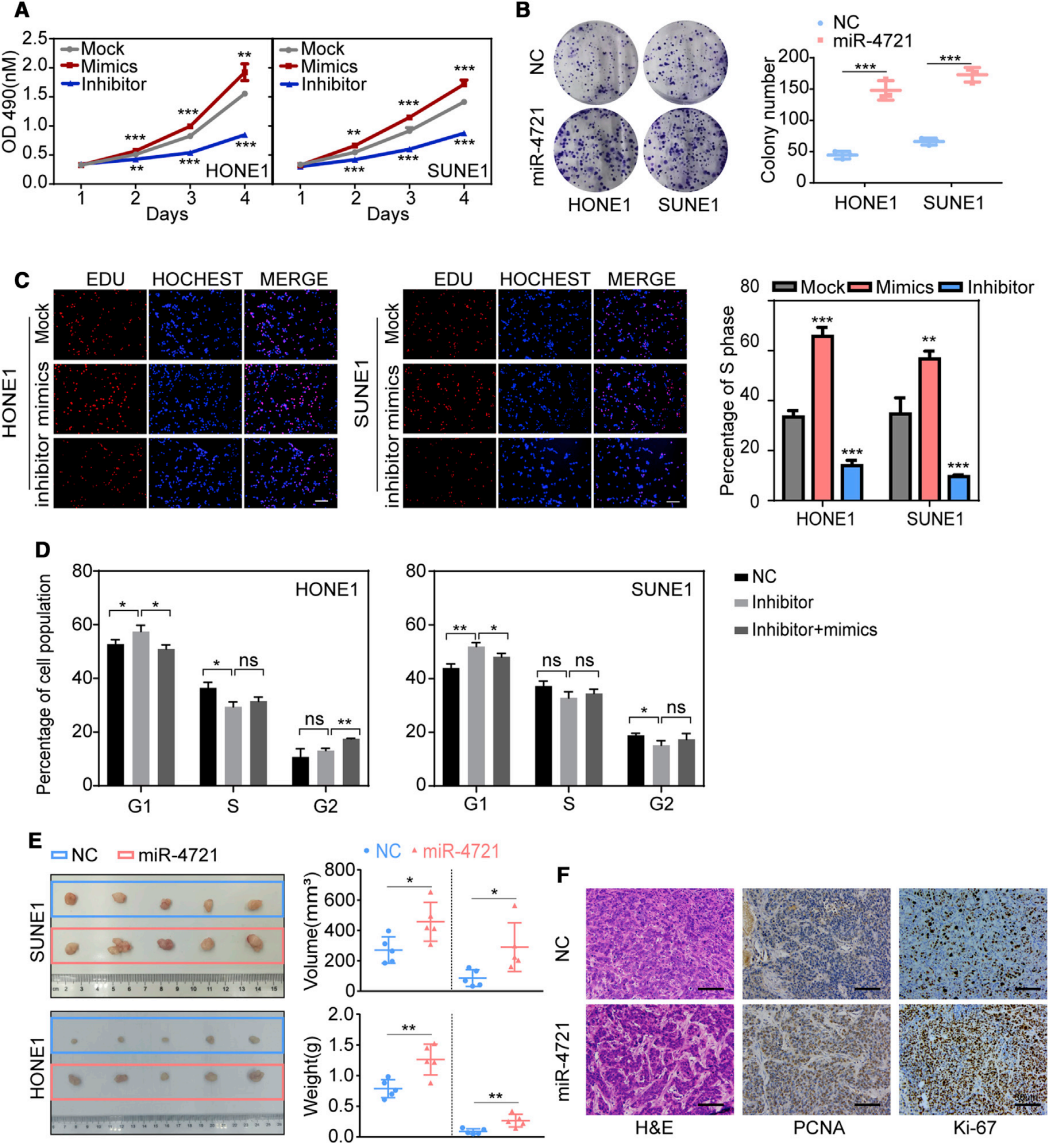
miR-4721 Promotes NPC Proliferation *In Vitro* and *In Vivo* (A and C) MTT assays (A) and EdU incorporation assays (C) were conducted after transfection with miR-4721 mimics or inhibitor. Student’s t test. Mean ± SD. ∗∗p < 0.01, ∗∗∗p < 0.001. Scale bars, 200 μm. (B) Colony formation assays were performed after transfection with lentiviral particles carrying the miR-4721 precursor or negative control. Student’s t test. Mean ± SD. ∗∗∗p < 0.001. (D) Cell cycle of HONE1 and SUNE1 cells transfected with miR-4721 inhibitors with/without mimics (n = 3). Student’s t test. Mean ± SD. ∗p < 0.05, ∗∗p < 0.01. (E) Xenograft tumors collected on day 15 after subcutaneous implantation of SUNE1-NC, SUNE1-miR-4721, HONE1-NC, and HONE1-miR-4721 cells on nude mice. Tumor volume and tumor weight were measured on day 15 (n = 5), Student’s t test. Mean ± SD. ∗∗p < 0.01, ∗p < 0.05. (F) Representative H&E staining as well as PCNA and Ki-67 IHC of primary tumor tissues are shown. Original magnification, ×200; scale bars, 30 μm. Mean ± SD (n = 3). ∗∗p < 0.01.

First, we explored the influence of miR-4721 on NPC cell proliferation *in vitro*. miR-4721 inhibitors or mimics were transiently transfected into HONE1 or SUNE1 cells, respectively. We performed MTT (3-(4,5-dimethylthiazol-2-yl)-2,5-diphenyltetrazolium bromide) (Figure 2A) and EdU (5-ethynyl-2′-deoxyuridine) incorporation (Figure 2C) assays to investigate the effects of miR-4721 on NPC cell proliferation. We then used lentiviral particles carrying the hsa-miR-4721 precursor to generate two stably transfected cell lines, HONE1-miR-4721 and SUNE1-miR-4721. The expression level of miR-4721 was measured by qRT-PCR after transfection (Figures S1A–S1C). A colony formation assay was performed with these two stably expressing miR-4721 cell lines (Figure 2B). These assays showed that overexpression of miR-4721 promotes cell proliferation *in vitro*.

Flow cytometry was used to determine whether miR-4721 affected the cell cycle of NPC cells. miR-4721 inhibitor transfection resulted in 3.33% and 5.24% increases of G_1_ phase cell numbers in the two cell lines, respectively (p < 0.05), while the S phase percentage decreased in HONE1 cells and the G_2_ phase percentage was decreased in SUNE1 cells accordingly, when compared with the controls. These results suggest that the miR-4721 inhibitor induced cell cycle arrest by blocking the cells in the G_1_ phase and led to cell number reductions in the S or G_2_ phase. Co-transfection of the miR-4721 inhibitor and mimics reversed the cell cycle arrest (Figure 2D; Figure S2A). These results demonstrate that the miR-4721 inhibitor decreases proliferation by inducing cell cycle arrest.

To fully confirm the role of miR-4721 in NPC carcinogenesis, we performed an *in vivo* tumor formation experiment by subcutaneously injecting SUNE1-NC or SUNE1-miR-4721 cells and HONE1-NC or HONE1-miR-4721 cells into nude mice. After 15 days, mice injected with SUNE1-miR-4721 or HONE1-miR-4721 had higher tumor burdens (Figure 2E) and displayed elevated Ki-67 and proliferating cell nuclear antigen (PCNA) expression in the overexpressed (oe-)miR-4721 group relative to the negative control (Figure 2F). In summary, our experiments confirm that miR-4721 promotes G_1_/S cell cycle transition and thus NPC proliferation, both *in vitro* and *in vivo*.

### *GSK3β* Is a Target Gene for miR-4721

**Figure 3:**
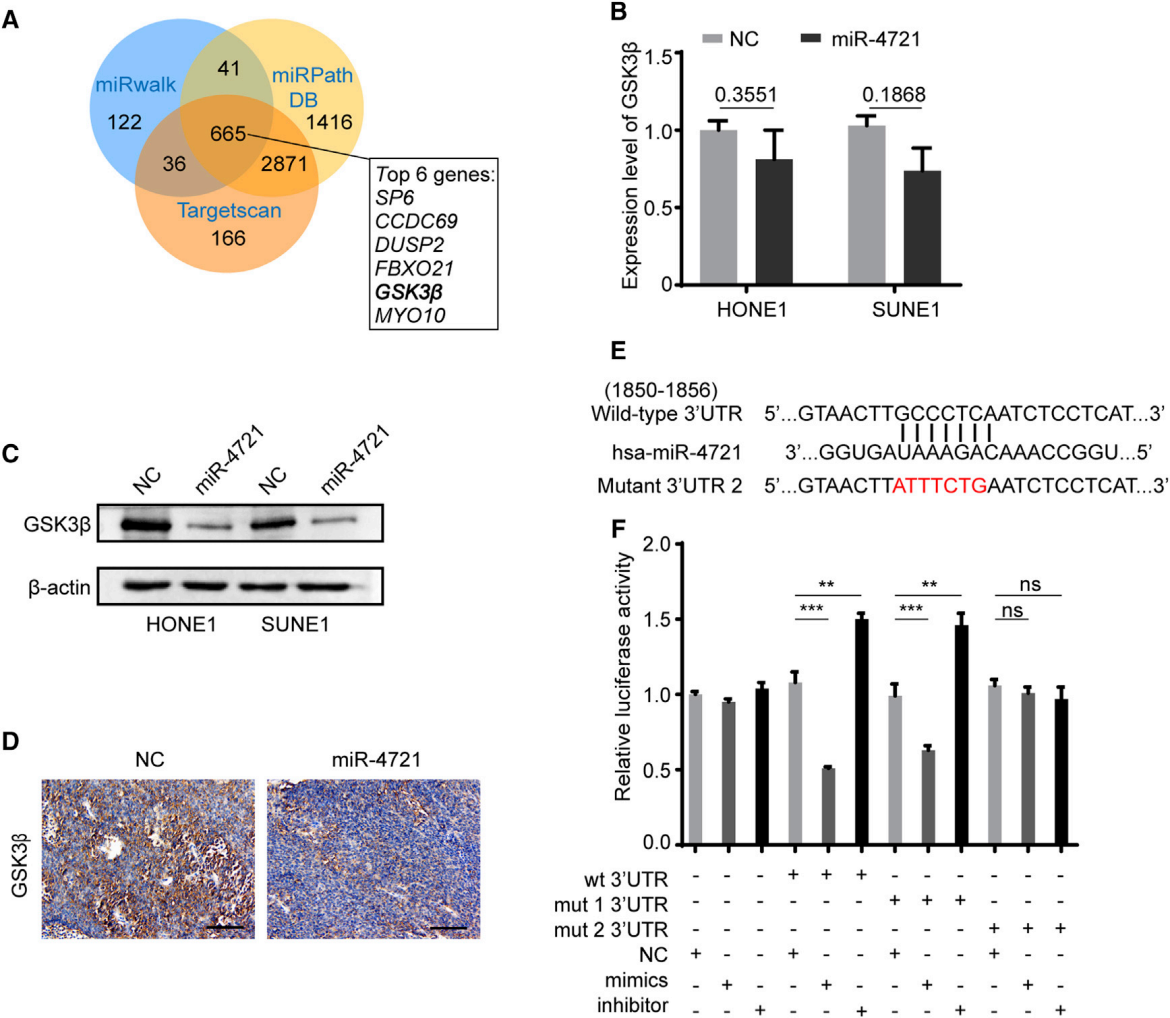
miR-4721 Directly Targets *GSK3β* to Activate the *WNT/β-catenin* Pathway (A) A Venn diagram shows the results of bioinformatics analysis of miR-4721 and its putative target genes through miRWalk, miRPathDB, and TargetScan. 665 common putative genes were found, and six top-scored genes are shown on the right. (B) We detected *GSK3β* expression by qRT-PCR in oe-miR-4721 NPC cells, normalized to GAPDH. Student’s t test. Mean ± SD. ns, not significant. (C) The protein level of *GSK3β* in oe-miR-4721 NPC cells. β-Actin was used as a loading control. Student’s t test. Mean ± SD. ∗p < 0.05. (D) We evaluated GSK3β expression by IHC in xenografts derived from NPC xenograft nude models. Scale bars, 30 μm. (E) Bioinformatics predictions of binding site by miR-4721 in the *GSK3β* 3′ UTR region. (F) A luciferase reporter assay was conducted to detect the combination between the seed region of miR-4721 and the 3′ UTR region of *GSK3β*. Mean ± SD. ∗p < 0.05, ∗∗p < 0.01, ∗∗∗p < 0.001. ns, not significant.

To learn more about the underlying mechanism of miR-4721 in NPC, we used the bioinformatics online tools TargetScan, miRWalk, and miRPathDB to predict the targets of miR-4721. A total of 665 genes were found by these three online tools (Figure 3A). Prediction results showed that the *GSK3β* 3′ UTR region contains two sites complementary with the seed sequences of miR-4721.

To explore the effects of miR-4721 on *GSK3β* expression, we overexpressed miR-4721 in HONE1 and SUNE1 cells and found that the protein level of *GSK3β* was downregulated, while its RNA level did not change when compared to the NC group (Figures 3B and 3C). We thus confirm that miR-4721 might be regulating *GSK3β* only at the post-transcriptional level. Immunohistochemistry (IHC) staining of xenograft tumor sections demonstrated that the upregulation of miR-4721 reduced the expression of GSK3β, which confirmed our deduction (Figure 3D).

Bioinformatics data indicated that the 3′ UTR region of *GSK3β* and the miR-4721 seed sequence are well matched (Figure 3E). To verify whether miR-4721 directly targets *GSK3β*, luciferase reporter assays were conducted by co-transfection of wild-type (WT) or mutant (mut 1 or mut 2) *GSK3β* 3′ UTR-containing luciferase reporter vectors with miR-4721 mimic/inhibitor. The luciferase activity of WT *GSK3β* 3′ UTR and mutant 3′ UTR (mut 1), but not that of mutant 3′ UTR (mut 2), was significantly modulated by miR-4721 mimic/inhibitor, but not by the control mimic/inhibitor (Figure 3F). This demonstrates that miR-4721 binds to the 3′ UTR region of *GSK3β* through site 2 and directly suppresses its expression.

### miR-4721 Promotes *β-Catenin* Transport to the Nucleus through *GSK3β*

**Figure 4:**
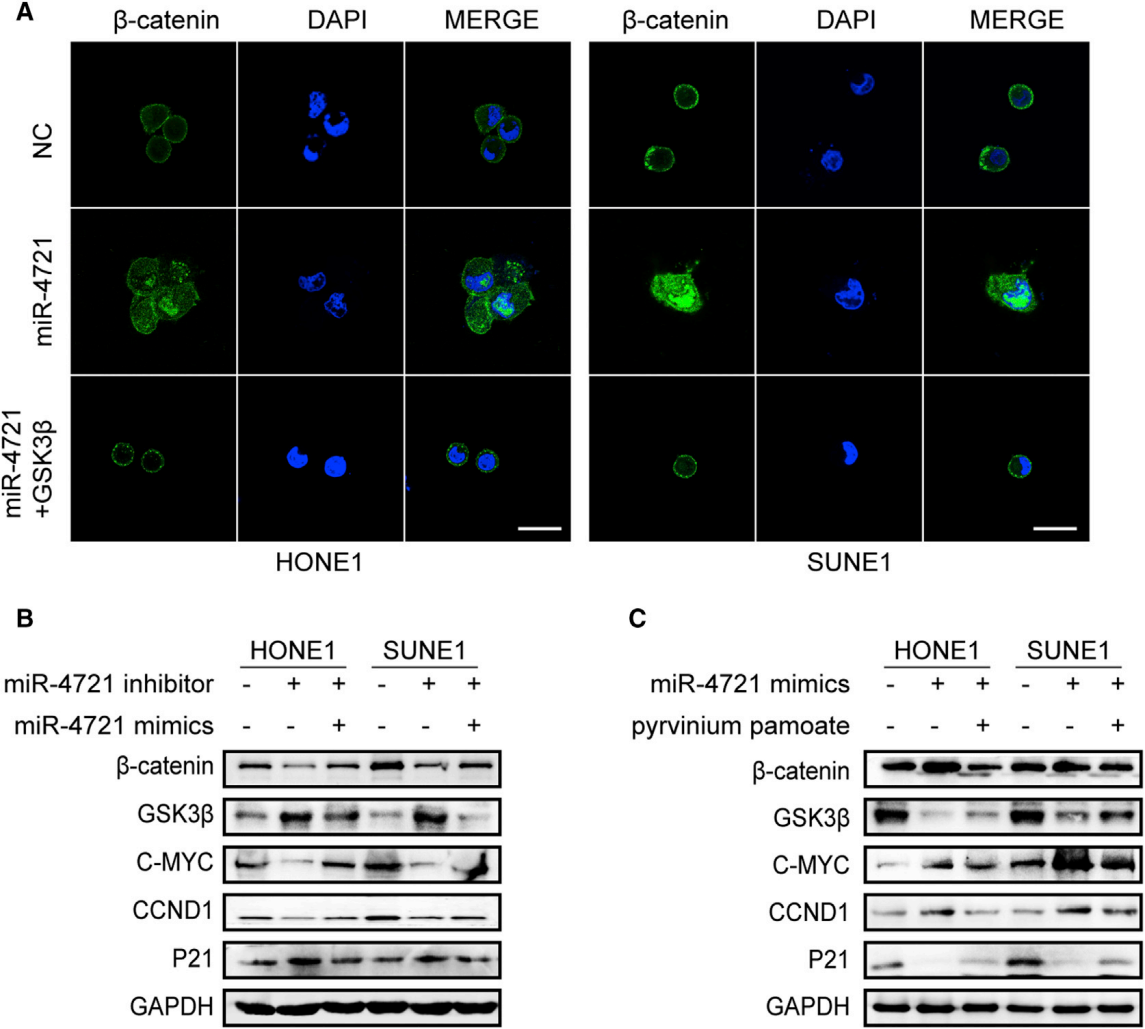
miR-4721 Leads to *β-catenin* Nuclear Translocation and Activates the *WNT*/*β-catenin* Signaling Pathway (A) Immunofluorescence staining of DAPI and *β-catenin* localization in miR-4721 mimics, miR-4721 mimics with *GSK3β*, and NC cells. Scale bars, 25 μm. (B) Expression of β-catenin, GSK3β, c-MYC, CCND1, and p21 were detected following transfection of miR-4721 inhibitors and/or mimics, HONE1 cells, and SUNE1 cells; GAPDH was used as a loading control. (C) Expression of β-catenin, GSK3β, C-MYC, CCND1, and p21 were detected after transfection with mock, mimics, and/or pyrvinium pamoate as indicated. GAPDH was used as a loading control.

miR-4721 mimics and/or *GSK3β* plasmids were transfected into HONE1 and SUNE1 cells, respectively, and *β-catenin* localization was detected using immunofluorescence staining. Results showed that more *β-catenin* was transported into the nucleus in the oe-miR-4721 group compared to the NC group. However, co-transfection with miR-4721 mimics and *GSK3β* plasmid decreased the transport of *β-catenin* into the nucleus (Figure 4A). These data suggest that miR-4721 promotes *β-catenin* transportation into the nucleus.

### miR-4721 Activates the *WNT*/*β-Catenin* Signaling Pathway

As an indispensable component of the *WNT*/*β-catenin* pathway, *GSK3β*, along with *APC* and *Axin*, forms a *β-catenin* degradation complex. To explore whether the downregulation of *GSK3β* by miR-4721 affects the *WNT*/*β-catenin* pathway, we detected the key genes after transfecting the cells with miR-4721 inhibitors and/or mimics. Western blot showed that β-catenin, c-MYC, and CCND1 were downregulated while GSK3β and p21 were upregulated when the cells were transfected with miR-4721 inhibitor, and these tendencies were reversed when transfected with miR-4721 inhibitor and mimics at the same time (Figure 4B). We then used a *WNT* pathway-specific inhibitor, pyrvinium pamoate, and found that it could eliminate miR-4721-initiated activation of the *WNT* pathway (Figure 4C). These results suggest that miR-4721 promotes carcinogenesis by directly activating the *WNT*/*β-catenin* pathway.

### *GSK3β* Overexpression Attenuates the Promotion of Proliferation and Cell Cycle Progression Caused by miR-4721

**Figure 5:**
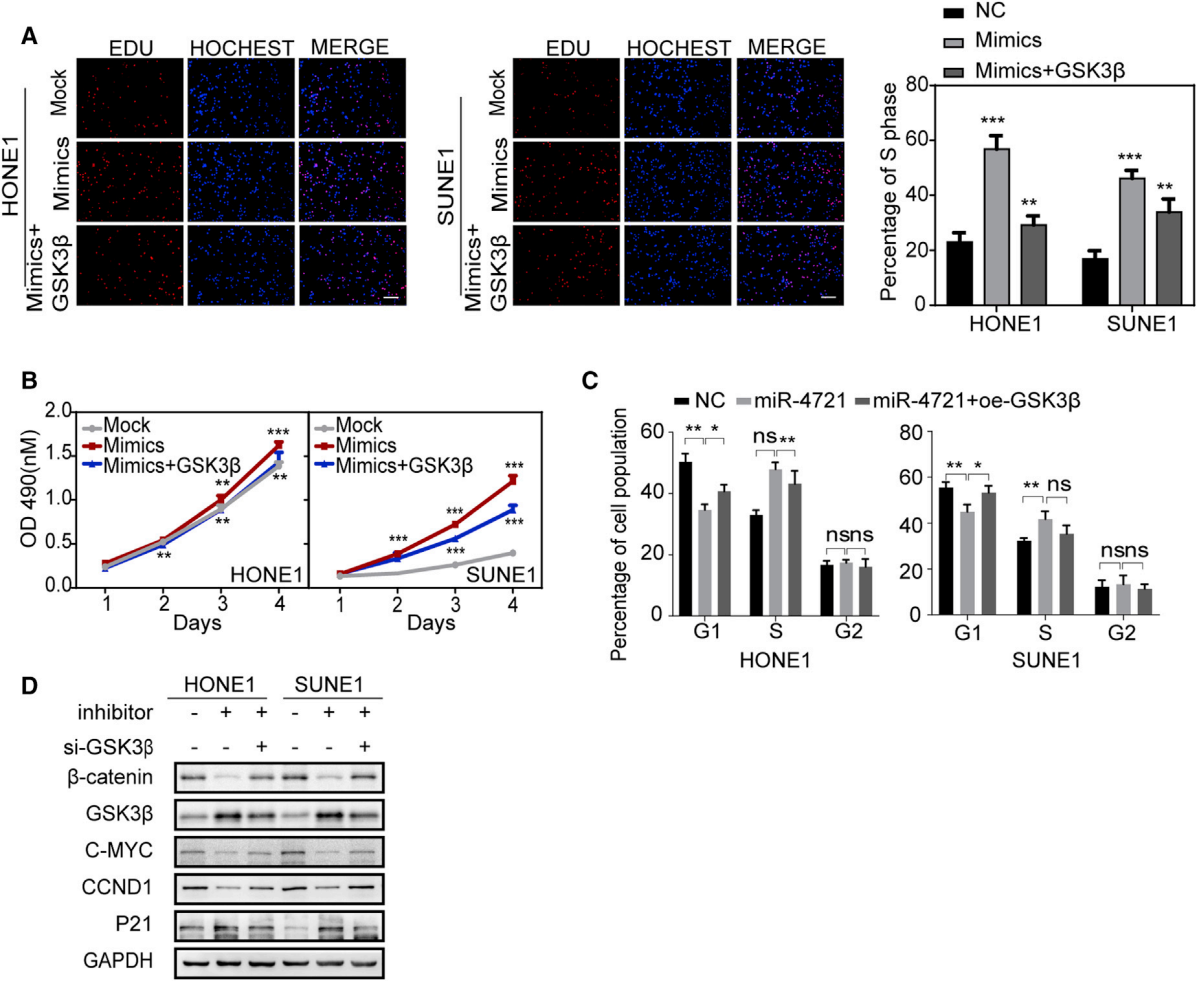
*GSK3β* Re-expression Rescued the Phenotypes Generated by miR-4721 (A and B) EdU incorporation assays (A) and MTT assays (B) were conducted after transfection with miR-4721 mimics with/without *GSK3β* plasmid. Scale bars, 200 μm. Mean ± SD. ∗p < 0.05, ∗∗p < 0.01, ∗∗∗p < 0.001. (C) Cell cycle of HONE1 and SUNE1 cells transfected with miR-4721 mimics with/without *GSK3β* plasmid (n = 3). Student’s t test. Mean ± SD. ∗p < 0.05, ∗∗p < 0.01. (D) *WNT* signaling and cell cycle regulators including β-catenin, GSK3β, c-MYC, CCND1, and p21 were detected by western blot after transfection with NC, miR-4721 inhibitor, and/or si-*GSK3β* as indicated. GAPDH was used as a loading control.

We then transiently transfected *GSK3β* and miR-4721 mimics together into NPC cells (Figure S1D), and we found that the promotion of cell proliferation by miR-4721 mimics was attenuated, as indicated by MTT and EdU incorporation assays (Figures 5A and 5B) assays, and it also decreased the transition from G_1_ to S in the cell cycle (Figure 5C; Figure S2B). These results suggested that overexpressed *GSK3β* attenuates NPC cell growth promotion induced by miR-4721.

In mechanism studies, we found that suppression of GSK3β by small interfering RNA (siRNA) directed against GSK3β (si-*GSK3β*) prevented the reduction of β-catenin, c-MYC, and CCND1, as well as the increases of p21 that were caused by the miR-4721 inhibitor (Figure 5D). This further confirms that miR-4721 activates *WNT*/*β-catenin* signaling through *GSK3β*.

### miR-4721 Is Upregulated by *Sp1*

**Figure 6:**
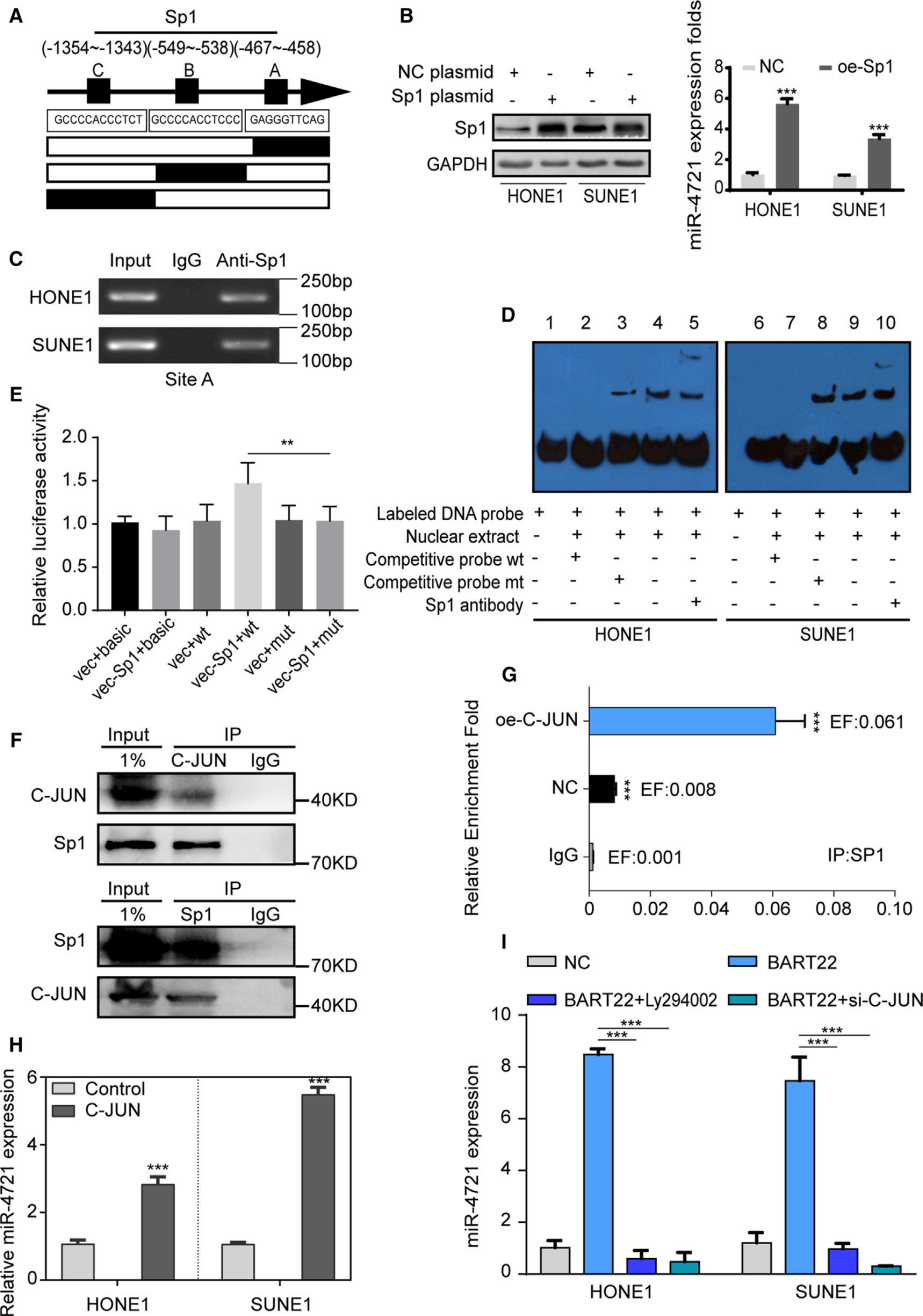
*Sp1* Binds to the Promoter Region of miR-4721, and EBV-miR-BART22 Induces miR-4721 through *PI3K*/*AKT*/*c-JUN*/*Sp1* (A) Schematic representation of the promoter regions of miR-4721 with the putative *Sp1* binding sites (site A, site B, and site C). (B) miR-4721 expression was detected by qPCR after *Sp1* plasmid was transfected in both HONE1 and SUNE1 cells. Student’s t test. Mean ± SD. ∗∗∗p < 0.001. (C) PCR gel showing amplification of *Sp1*-binding site A after a ChIP assay using antibody against *Sp1*. (D) EMSA results are shown from nuclear proteins extracted from HONE1 and SUNE1 cells after incubation with individual DIG-ddUTP-labeled oligonucleotide probes. A labeled wild-type probe was incubated without (lanes 1 and 6) or with (lanes 4 and 9) cell nuclear proteins in the absence or presence of an unlabeled probe (lanes 2, 3, 7, and 8). An unlabeled wild-type probe (lanes 2 and 7) and mutant *Sp1* probe (lanes 3 and 8) were used to compete with *Sp1* binding, each at 100-fold excess. A supershift assay (lanes 5 and 10) was performed using an anti-*Sp1* antibody. (E) A luciferase reporter assay was used to determine *Sp1* direct targeting of the miR-4721 promoter region. Mean ± SD. ∗p < 0.05, ∗∗p < 0.01, ∗∗∗p < 0.001. ns, not significant. (F) Co-immunoprecipitation of total cell and the cytoplasmic fraction for *Sp1* in SUNE1. Lysates were immunoprecipitated with *c-JUN* antibody or control IgG and detected with *Sp1* antibody on western blot, then immunoprecipitated with *Sp1* antibody or control IgG and detected with *c-JUN* antibody on western blot. (G) ChIP assay was conducted in both the oe-c-JUN group and the NC group. DNA-protein complexes were immunoprecipitated using anti-Sp1 or IgG antibodies. (H) miR-4721 expression was detected by qPCR in oe-c-JUN and control cells. (I) miR-4721 expression was detected in oe-BART22, oe-BART22 and Ly294002, oe-BART22 and si-*c-JUN*, and the NC group both in HONE1 and SUNE1 cells. Student’s t test. Mean ± SD. ∗∗∗p < 0.001.

To find out how miR-4721 expression was regulated, we used several bioinformatics software programs (UCSC, PROMO, and TFSEARCH) to predict potential regulatory factors of miR-4721. We identified three potential *Sp1*-binding sites at −467 to −458, −549 to −538, and −1354 to −1343 inside the miR-4721 promoter region and named them site A, site B, and site C, respectively (Figure 6A). We first used *Sp1* plasmid to upregulate *Sp1* expression in HONE1 and SUNE1 cells. Next, qRT-PCR analysis showed that miR-4721 expression was markedly increased after *Sp1* overexpression (Figure 6B), suggesting that *Sp1* is an upstream regulator of miR-4721.

To found out whether these sites were actual *Sp1-*binding sites, we conducted chromatin immunoprecipitation (ChIP) assays. It was verified that Sp1 was recruited to binding site A, while site B or site C was not functional (Figure 6C). We then performed electrophoresis mobility shift assays (EMSAs) to verify this combination. Digoxigenin-2′,3′-dideoxyuridine-5′-triphosphate (DIG-ddUTP)-labeled *Sp1* probe was incubated to form a shifted band (lanes 1 and 6), and it was incubated with nuclear proteins extracted together (lanes 4 and 9), whereas the band disappeared when an unlabeled competitive probe was added for binding competition (lanes 2 and 7). No band variations were observed when mutated A was added (lanes 3 and 8) (Figure 6D). The EMSA results demonstrate that *Sp1* binds to binding site A in the promoter region of miR-4721. Furthermore, an upregulation of the luciferase activity was observed when *vec-Sp1* was combined with the WT binding sequence, while the effect was abolished when site A was mutated (Figure 6E). These data confirmed that *Sp1* binds to the promoter region of miR-4721 to promote its transcription.

### EBV-miR-BART22 Induces miR-4721 Expression through the *PI3K*/*AKT*/*c-JUN*/*Sp1* Signaling Axis

In a previous study, we found that EBV-miR-BART22 could activate the *PI3K*/*AKT* pathway and upregulate c-JUN expression.^24^ It is known that c-JUN interacts with Sp1 in certain tumors,^25^, ^26^, ^27^ and c-JUN acts as a synergy factor to enhance the transcriptional activity of Sp1.^28^^,^^29^ Thus, we hypothesized that EBV-miR-BART22 induces miR-4721 through c-JUN/Sp1 interaction, and upregulated c-JUN facilitates the transcriptional regulationship between *Sp1* and miR-4721. To verify our hypothesis, co-immunoprecipitation (coIP) was performed. coIP was done and included the whole cell and the cytoplasmic fraction of Sp1 in SUNE1. Lysates immunoprecipitated with c-JUN or immunoglobulin G (IgG) were detected with western blot using Sp1 antibody. Then, we repeated the immunoprecipitation using Sp1 and IgG, which used c-JUN for detection (Figure 6F). Results show that c-JUN interacts with Sp1 in NPC cells. Subsequently, a ChIP assay was conducted both in the oe-c-JUN group and the NC group, and DNA-protein complexes were immunoprecipitated using anti-Sp1 or IgG antibodies. The relative fold enrichment of IP-SP1 was 0.061 in the oe-c-JUN group and 0.008 in the NC group (Figure 6G, p < 0.001), which demonstrated that c-JUN facilitates the transcriptional activity of *Sp1*. Also, c-JUN expression was positively related to miR-4721 expression in NPC cells (Figure 6H; Figure S1E). Following that, we detected miR-4721 expression separately in four groups: NC, BART22 mimics, BART22 with Ly294002, and BART22 with si-*c-JUN*. Results showed that both Ly294002 and si-*c-JUN* decreased BART22-induced upregulation of miR-4721 (Figure 6I). These results demonstrate that EBV-miR-BART22 regulates miR-4721 expression through the *PI3K*/*AKT*/*c-JUN*/*Sp1* signaling axis.

### Pathoclinical Features of miR-4721 Expression and Its Correlation with *GSK3β*

**Figure 7:**
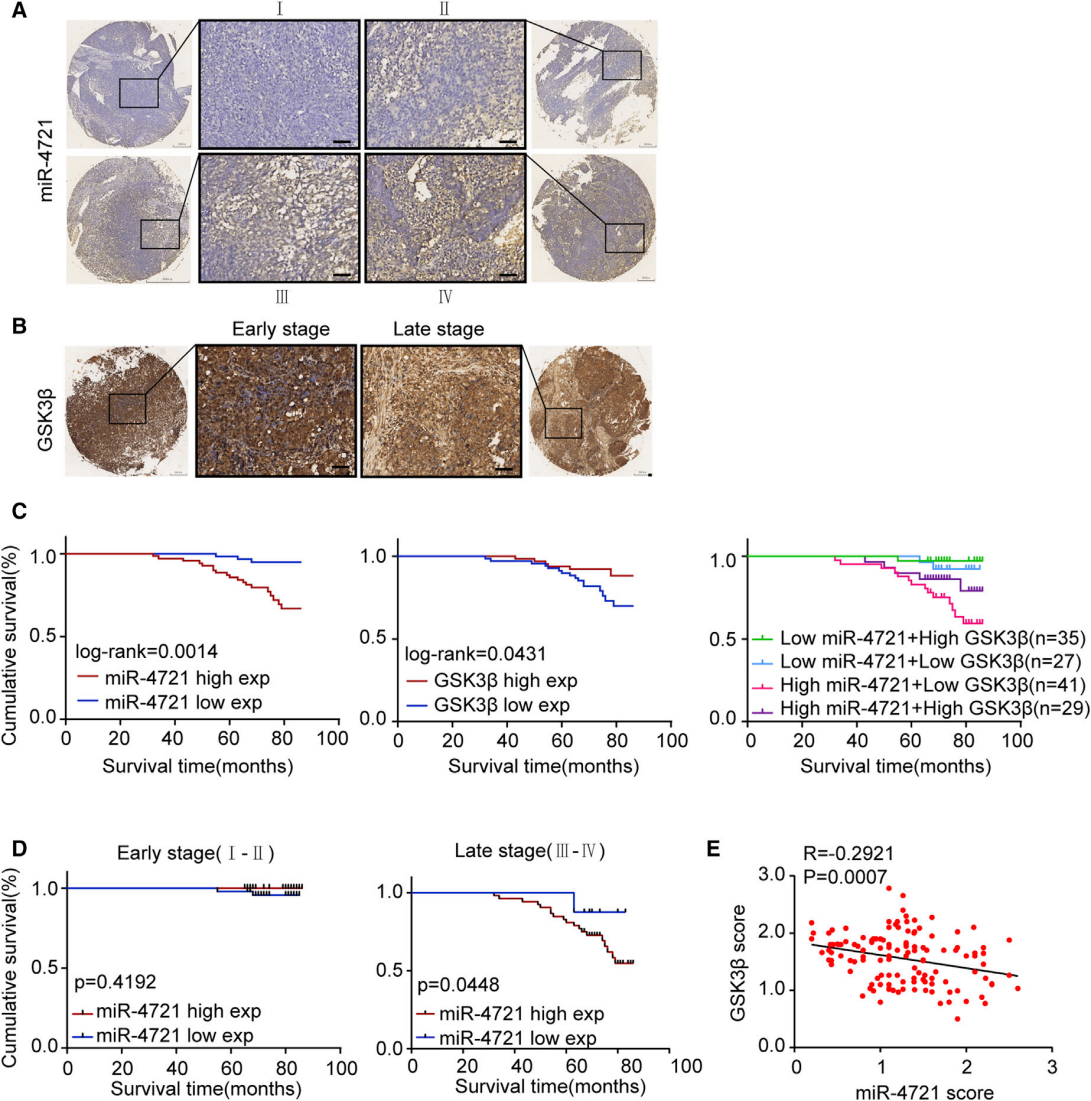
Pathoclinical Features of miR-4721 Expression and Its Correlation with *GSK3β* (A) *In situ* hybridization was conducted to detect the miR-4721 expression in tissue microarrays of 132 NPC specimens. Significant upregulation of miR-4721 in advanced clinical stages (III–IV) compared with early clinical stages (I–II) is shown. (B) IHC staining of *GSK3β* in tissue microarrays. (C) Kaplan-Meier survival analysis of overall survival of 132 NPC patients based on miR-4721 and GSK3β different expression levels. The log-rank test was used to calculate p values. Right lane: Kaplan-Meier survival analysis of overall survival in 132 NPC patients on the basis of different expression level combinations of miR-4721 and GSK3β. (D) Kaplan-Meier survival analysis of overall survival of early-stage and late-stage NPC patients based on miR-4721 expression level. The log-rank test was used to calculate p values. (E) Correlations between miR-4721 and GSK3β expression levels were calculated. Two-tailed Spearman’s correlation analysis. Mean ± SD (R = −0.2921, p = 0.0007).

To detect the expression of miR-4721 in clinical NPC tissues, an *in situ* hybridization assay was conducted in tissue microarrays of 132 NPC specimens. It can be confirmed that the expression levels of miR-4721 and GSK3β are associated with the overall survival time of NPC patients (Figures 7A and 7B). Clinical-associated features are presented in Tables 1 and 2. No significant correlations were found between miR-4721/GSK3β and patient age, sex, or metastasis (M) stage. However, there is a significant correlation between miR-4721 expression level and patient clinical stage (p = 0.0001), node (N) stage (p = 0.0002), and recurrence (p = 0.003). Also, survival analysis showed that patients with lower miR-4721 expression had a better prognosis than did the group with higher miR-4721 expression (p = 0.0014), which was exactly the opposite result compared to the GSK3β group (p = 0.0431). The best survival prognosis was for NPC patients with low miR-4721 and high GSK3β expression, when compared to the other three groups (Figure 7C). We did not find a prognostic difference between miR-4721 high and low expression groups in early-stage NPC patients. However, in late-stage NPC patients, the miR-4721 low expression group tended to have a better prognosis (Figure 7D). Levels of miR-4721 were significantly higher and GSK3β was significantly lower in late-stage compared to early-stage NPC specimens, based on fluorescence *in situ* hybridization (FISH) analysis-grouped scores. As such, GSK3β expression was negatively correlated with miR-4721 expression (R = −0.2921, p = 0.0007; Figure 7E).

**Table 1:** Correlation between the Clinicopathologic Characteristics and Expression of miR-4721 in NPC

Characteristics	n	miR-4721 Expression		p Value
		High	Low	
Age, years				
<50	74	38	36	0.7294
≥50	58	32	26	
Sex				
Male	101	52	49	0.2688
Female	31	18	13	
Clinical stage				
I–II	72	18	54	0.0001
III–IV	60	52	8	
N stage				
N0–N1	36	9	27	0.0002
N2–N3	96	61	35	
M stage				
M0	130	68	62	0.3176
M1	2	2	0	
Recurrence				
Yes	60	38	22	0.003
No	72	32	40	

p values were determined by a t test. NPC, nasopharyngeal carcinoma; N stage, node stage; M stage, metastasis stage.

**Table 2:** Correlation between the Clinicopathologic Characteristics and Expression of GSK3β in NPC

Characteristics	n	GSK3β Expression		p Value
		High	Low	
Age, years				
<50	74	36	38	0.6826
≥50	58	28	30	
Sex				
Male	101	49	52	0.9578
Female	31	15	16	
Clinical stage				
I–II	72	44	28	0.0001
III–IV	60	20	40	
N stage				
N0–N1	36	23	13	0.0144
N2–N3	96	41	55	
M stage				
M0	130	63	67	0.9346
M1	2	1	1	
Recurrence				
Yes	60	22	38	0.0022
No	72	42	30	

p values were determined by a t test. NPC, nasopharyngeal carcinoma; N stage, node stage; M stage, metastasis.

## Discussion

Previous studies on EBV-miR-BART22 mainly focused on its expression and basic phenotype in gastric cancer and liver transplantation patients.^30^^,^^31^ Our recent study has presented a novel mechanism of EBV-miR-BART22 in inducing cisplatin (DDP) chemoresistance of NPC by stimulating tumor stemness and the epithelial-to-mesenchymal transition (EMT) signal. However, there are other molecules involved in tumor growth of EBV-miR-BART22 that have yet to be identified. To further understand the character of EBV-miR-BART22 in NPC progression, we detected the differentially expressed miRNAs between oe-EBV-miR-BART22 cells and control cells, using a miRNA chip and qRT-PCR. We found a positive relationship between EBV-miR-BART22 and miR-4721, which suggested that miR-4721 might serve as a downstream effector of EBV-miR-BART22.

To further investigate the role of miR-4721, we first examined its expression in NPC cells and immortalized nasopharyngeal epithelial cells. Data showed upregulated miR-4721 expression in NPC cells. Functional assays showed that overexpression of miR-4721 promotes proliferation both *in vitro* and *in vivo*, while the miR-4721 inhibitor induces G_1_ phase arrest by blocking cells in the G_1_ phase and leads to cell number reductions of the S or G_2_ phase. Follow-up experiments have shown that G_1_ phase-specific factors CCND1 and c-MYC were downregulated when cyclin-dependent kinase inhibitor p21 was elevated in the miR-4721 inhibitor group. These data suggested that miR-4721 serves as an oncomiR in NPC.

*GSK3β* functions as a negative regulator of *β-catenin*, along with *APC* and *Axin* in the *WNT* signaling path.^32^^,^^33^ According to previous reports, *GSK3β* is inactivated, with concomitant nuclear *β-catenin* accumulation, in most NPC specimens.^34^^,^^35^ Downregulation of *GSK3β* drives *β-catenin* nucleus transport and accumulation, and it activates downstream effectors, such as *c-MYC* and *CCND1*, to promote cell cycle progression and proliferation.^36^, ^37^, ^38^ Bioinformatics websites TargetScan and miRDB predicted that there are two putative miR-4721 binding sites in the 3′ UTR region of *GSK3β*. We detected *GSK3β* expression in miR-4721 overexpressing cells, using qRT-PCR and western blot. Results showed that miR-4721 decreases *GSK3β* protein expression rather than its mRNA expression, which suggests that miR-4721 regulates *GSK3β* at a post-transcriptional level. A luciferase assay was then used to confirm the combined effectiveness of miR-4721 and the 3′ UTR of *GSK3β*. Results showed that after the mutation of binding site 1, the mimic and inhibitor of miR-4721 still have a regulatory effect on the target gene, whereas the regulatory effect was gone after binding site 2 mutation. These results showed that miR-4721 targets *GSK3β* through site 2 and represses its expression at the post-transcriptional level. Subsequently, immunofluorescence staining showed that overexpression of miR-4721 promotes accumulation of β-catenin, which is consistent with our hypothesis that miR-4721 targets *GSK3β* to promote β-catenin nuclear translocation. Finally, overexpressed *GSK3β* diminished the upregulatory effect of miR-4721 overexpression on β-catenin, c-MYC, and CCND1. Taken together, miR-4721 targets *GSK3β* to activate the *WNT*/*β-catenin* signal, and it functions as an oncogene in NPC growth.

In previous studies, miRNAs have been widely reported to be regulated by various transcription factors.^39^, ^40^, ^41^, ^42^ *Sp1* belongs to a famous *Sp* transcription family, which also includes *Sp2*, *Sp3*, *and Sp4*, and it has been importantly proven to modulate essential biological processes such as cell growth,^43^ differentiation,^44^ and apoptosis and carcinogenesis.^45^ For many cancer patients, elevated Sp1 expression is considered as an adverse prognostic factor.^46^^,^^47^ Bioinformatics analysis was conducted and several putative *Sp1-*binding sites for miR-4721 were predicted. qRT-PCR results showed that *Sp1* upregulates miR-4721 expression. Luciferase, EMSA, and ChIP assays were subsequently conducted to demonstrate the association of *Sp1* with the promoter region of miR-4721. These results showed that *Sp1* promotes miR-4721 transcription by directly binding to its promoter region. To clarify the regulatory relationship among EBV-miR-BART22, *Sp1*, and miR-4721, we examined the downstream effectors of EBV-miR-BART22 in a previous work;^24^ unfortunately we found no signs of direct regulating relationships between EBV-miR-BART22 and *Sp1.* There could be another mechanism to explain how miR-4721 was regulated.

*c-JUN* is known as an important transcription factor involved in many carcinogenic processes, especially in cell cycle progression,^48^ anti-apoptotic activity,^49^ and cell proliferation,^50^ and it serves as a downstream effector of the *PI3K*/*AKT* signal.^51^^,^^52^ It was reported to be involved in many miRNAs’ regulation as a transcription factor, such as miR-374a^53^^,^^54^ and miR-3188.^55^ Our previous study showed that *c-JUN* serves as a downstream effector of the BART22/*PI3K*/*AKT* signal. During our early study, we demonstrated that *c-JUN* expression was positively related to miR-4721 expression in NPC cells. Using the bioinformatics websites PROMO and JASPAR, we found two potential binding sites between *c-JUN* and miR-4721; unfortunately, however, a ChIP-qPCR assay showed that the results of two potential binding sites were all negative. Thus, we proved that miR-4721 was not directly regulated by c-JUN, and the mechanism of miR-4721 regulation needs further exploration. It is well established that *Sp1* interacts with *c-JUN* in several cancers,^56^ and *c-JUN* acts synergistically with *Sp1* to activate downstream oncogenes.^57^ In subsequent research, we demonstrated that *c-JUN* interacts with *Sp1* in NPC, using a coIP assay. Also, oe-*c-JUN* in NPC cells leads to higher transcriptional activity of SP1, which brings up the suggestion that c-JUN facilitates the regulation between SP1 and miR-4721 by interacting with SP1. Further experiments showed that oe*-c-JUN* upregulates miR-4721 expression while the PI3K inhibitor ly294002 and si-*c-JUN* can both reverse the increase of miR-4721 induced by EBV-miR-BART22. Collectively, we demonstrate that miR-4721 is induced by EBV-miR-BART22 through the *PI3K*/*AKT*/*c-JUN*/*Sp1* signaling axis.

We then performed an *in situ* hybridization assay to examine miR-4721 levels in NPC tissue. Results show that miR-4721 is highly expressed in late-stage NPC tissues and that it is negatively correlated with GSK3β expression and NPC patient prognosis. These observations demonstrate the significance of abnormal miR-4721 in NPC pathogenesis and reveal the underlying value of miR-4721 as a prognostic indicator in NPC.

In summary, upregulated miR-4721 was shown as an unfavorable factor, promoting NPC pathogenesis. We showed that miR-4721 is induced by EBV-miR-BART22 through the *PI3K*/*AKT*/*c-JUN*/*Sp1* signaling axis to target *GSK3β*, which activates the *β-catenin*-stimulated cell cycle signal and thus enhance the tumorigenic capacity of NPC (Figure S2C). Our study reveals a novel mechanism for miR-4721 as a miRNA involved in NPC progression and clarifies the regulatory relationships between EBV-miR-BART22 and miR-4721 for the first time.

## Materials and Methods

### Tissue Specimens

Twenty NPC and 15 healthy nasopharyngeal specimens were obtained (Nanfang Hospital, Guangzhou, China) after diagnosis. All samples were immediately stored in liquid nitrogen after removal.

### IHC Staining

GSK3β, Ki-67, PCNA, CCND1, and c-MYC expression were detected by IHC staining. We performed the streptavidin-peroxidase-conjugated method according to the manufacturer’s instructions. Two pathologists were invited to examine IHC tissue sections. The details of the antibodies used are listed in Table S3.

### *In Situ* Hybridization

*In situ* hybridization was carried out by Bioscience (Guangzhou, China) to examine miR-4721 expression level in 132 paraffin-embedded NPC specimens. A diaminobenzidine (DAB) substrate kit (AxyBio, Guangzhou, China) was used to detect positive staining.

### Cell Culture and Transfection

We obtained four NPC cell lines (5-8F, CNE1, HONE1, and SUNE1) and NP69 from the Cancer Research Institute of Southern Medical University (Guangzhou, China). RPMI 1640 (Invitrogen) supplemented with 10% fetal bovine serum (FBS; HyClone, Invitrogen) was used for NPC cell culturing. Keratinocyte serum-free medium (KSFM) supplemented with epidermal growth factor (Invitrogen, Carlsbad, CA, USA) was used for NP69 culturing. Cells were incubated in 95% air and 5% CO_2_ at 37°C.

EBV-miR-BART22, miR-4721 mimics, inhibitors, and siRNAs for *c-JUN*, *GSK3β*, and their corresponding negative controls were obtained from RiboBio (Guangzhou, China). *Sp1* and *GSK3β* plasmids were obtained from Vigene Biosciences (Jinan, China). *WNT* inhibitor pyrvinium pamoate was purchased from MedChemExpress (Shanghai, China). Ly294002 was obtained from Sigma- Aldrich). NPC cells were plated into a six-well plates at 30%–50% confluence, 24 h before transfection. Plasmids, siRNAs, or inhibitors were transfected into NPC cells at a final concentration of 50 nmol/L while mimics were transfected into cells at a final concentration of 5 nmol/L using Lipofectamine 2000 (Invitrogen, Guangzhou, China) in serum-free conditions. Six hours later, the medium was replaced by RPMI 1640. Cells were collected 24–72 h afterward for further experiments.

### Western Blot

Western blot was performed according to western blot protocol (https://www.westernblotprotocol.com). Antibodies included anti-β-catenin, GSK3β, c-MYC, CCND1, p21, Sp1, c-JUN, GAPDH, and β-actin. Antibodies used are listed in Table S3.

### Cell Proliferation and Colony Formation Assays

The MTT assay was conducted to detect cell viability according the manufacturer’s protocol (Sigma-Aldrich). The optical density (OD) value was measured at 490 nm. NPC cells were seeded at a density of 100 cells/well for colony formation. After 2 weeks of culturing, cells were washed with PBS and stained with hematoxylin solution. Colonies composed of more than 50 cells in a well were counted. All experiments were repeated at least three times.

### Cell Cycle Analysis and EdU Incorporation Assay

The cell cycle analysis was conducted using cell a cycle and apoptosis kit (Leagene Bio, Beijing, China) according to the manufacturer’s protocol. Cells were transferred to serum-free medium for 24 h before testing to synchronize. For the EdU assay, proliferating NPC cells were examined using the Cell-Light EdU Apollo 488 or 567 *in vitro* imaging kit (RiboBio).

### *In Vivo* Tumorigenesis in Nude Mice

*In vivo* experiments were approved by the Animal Care and Use Committee of Southern Medical University and were performed in accordance with the National Institute of Health *Guide for the Care and Use of Laboratory Animals*. The research was approved by the Ethics Committee of Shanghai Outdo Biotech (control no. YB M-05-02). A total of 5 × 10^6^ logarithmically growing NPC cells transfected with miR-4721 or the control (n = 5 per group) in 0.1 mL of Hanks’ solution were subcutaneously injected into the mice (BALB/c, nu/nu, 4 weeks old, male). After 15 days, the mice were sacrificed and tumor tissues were excised and weighed.

### miRNA Array following Overexpressed EBV-miR-BART22

miRNA array was carried out by Gene Company (Shanghai, China). The Affymetrix Gene Chip Micro 2.0 Array (Affymetrix, Santa Clara, CA, USA) was used for universal miRNA coverage. Total RNA was isolated from oe-EBV-miR-BART22 and control cells. Statistical analysis was carried out using the open-source R software.

### Luciferase Reporter Assay

*GSK3β* was predicted to be directly regulated by miR-4721 using TargetScan, miRPathDB, and miRWalk. The WT 3′ UTR or mutant 3′ UTR was cloned into psiCHECK-2 vectors. The WT or mutant 3′ UTR vector was co-transfected with miR-4721 mimics/inhibitors or a non-specific control into cells. Luciferase activity was measured 48 h after transfection using the Dual-Luciferase reporter assay system (Promega, Madison, WI, USA). The sequences of the primers used in the luciferase activity reporter assay are listed in Table S2.

### ChIP

The UCSC, PROMO, and TFSEARCH bioinformatics software predicted the putative *Sp1*-binding sites on the miR-4721 promoter region. DNA-protein complexes were immunoprecipitated from HONE1 and SUNE1 cells using a ChIP kit (Thermo Fisher Scientific, Waltham, MA, USA) and anti-Sp1 or IgG (Cell Signaling Technology, Danvers, MA, USA) antibodies. qRT-PCR analysis and PCR analysis were used to measure the enrichment of the miR-4721 promoter region. The primers used in the ChIP assay are listed in Table S1.

### EMSA Analysis

Binding of *Sp1* to the miR-4721 promoter was detected using an EMSA kit (Roche Diagnostics, Basel, Switzerland) according to the manufacturer’s instructions. Signals were recorded using a BioSens gel imaging system (Biotop, Shanghai, China). EMSA analysis was performed at Biosense Bioscience (Guangzhou, China). The sequences of the probes used in the EMSA assay are listed in Table S4.

### CoIP

coIP was carried out using a Pierce coIP kit (Thermo Scientific, USA). Briefly, total proteins were extracted and quantified. A total of 1,000 μg of protein in 400 μL of supernatant was incubated with 10 μg of anti-Sp1, anti-c-JUN, or anti-IgG antibodies for 12 h at 4°C. Beads were washed, eluted in sample buffer, and boiled for 5 min at 95°C. Immune complexes were subjected to western blot analysis. Anti-IgG was used as a negative control.

### Statistical Analysis

Statistical analyses were performed with the SPSS 24.0 statistical software package (SPSS, Chicago, IL, USA). Data are expressed as the mean ± SD from at least three independent experiments. Comparisons between two groups were performed using a Student’s t test, one-way analysis of variance (ANOVA) for multiple groups, and a parametric generalized linear model with random effects for tumor growth. Survival analysis was performed using the Kaplan-Meier method. All statistical tests were two-sided. Statistical significance is designated as follows: ∗p < 0.05, ∗∗p < 0.01, and ∗∗∗p < 0.001.

### Data Availability

All relevant data are available from the authors.

## Author Contributions

M.Z., W.F., and Z. Liu conceived the study and supervised and coordinated all aspects of the work; Z.T., W.C., Y.X., and X. Lin designed the research, wrote the paper, and prepared figures and tables; X. Liu, Y. Li, and Y. Liu performed experiments, interpreted data, and prepared figures and tables; Z. Luo contributed analytical tools. All authors read and approved the manuscript.

## Conflicts of Interest

The authors declare no competing interests.

## Supplemental Information

Document S1. Figures S1 and S2 and Tables S1–S4

Document S2. Article plus Supplemental Information
