# Supplementary material for: miR-4721, Induced by EBV-miR-BART22, Targets GSK3β to Enhance the Tumorigenic Capacity of NPC through the WNT/β-catenin Pathway
Source: Mol Ther Nucleic Acids. 2020 Sep 23;22:557–71. doi: 10.1016/j.omtn.2020.09.021 (PMC7566007; doi:10.1016/j.omtn.2020.09.021)
Supplement: Document S1. Figures S1 and S2 and Tables S1–S4 [file mmc1.pdf]

OMTN, Volume *22*

Supplemental Information

miR-4721, Induced by EBV-miR-BART22, Targets *GSK3β* to Enhance the Tumorigenic Capacity of NPC through the *WNT/β-catenin* Pathway

ZiBo Tang, WeiFeng Chen, Yan Xu, Xian Lin, Xiong Liu, YongHao Li, YiYi Liu, ZhiJian Luo, Zhen Liu, WeiYi Fang, and MengYang Zhao

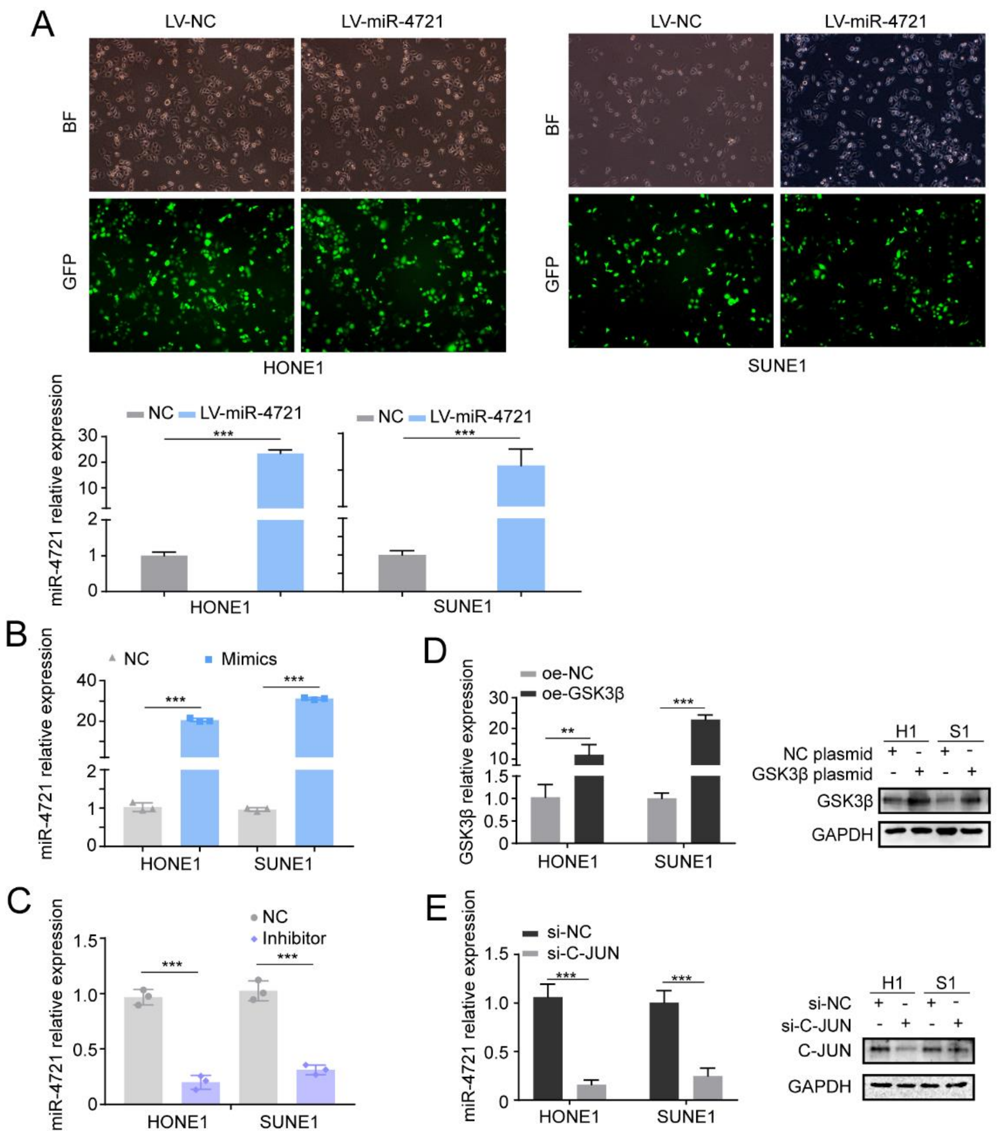

Figure S1. Expression level of miR-4721 after mimics, inhibitor, and lentiviral transfection.

(A) NPC cells (HONE1, SUNE1) transfected with miR-4721 lentiviral (GFP) were captured after 72h using a fluorescence microscope. miR-4721 relative expression levels were detected by q-PCR (****P* <0.001). (B-C) miR-4721 expression levels after transfection with mimics and inhibitor. Student's t-test. mean± SD (****P* <0.001). (D) Detection of GSK3β transfection efficiency on RNA and protein level. Student's t-test. mean± SD (***P* <0.01, ****P* <0.001). (E) Detection of miR-4721 and C-JUN expression after C-JUN knock down. Student's t-test. mean± SD (****P* <0.001).

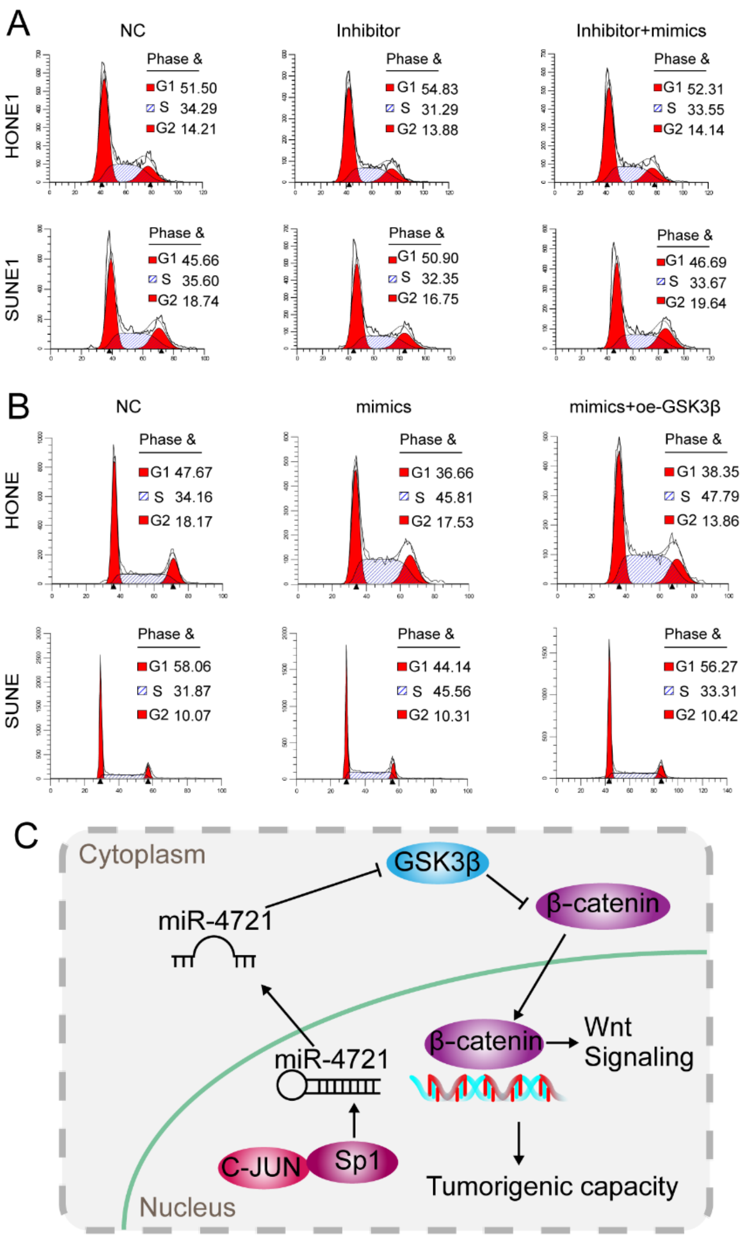

Figure S2. miR-4721 facilitates cell-cycle progression while *GSK3β* overexpression rescued it. (A) Cell cycle of HONE1 and SUNE1 cells transfected with miR-4721 inhibitors with/without mimics were determined by PI staining and flow cytometry (n=3 per group). (B) Cell cycle of HONE1 and SUNE1 cells transfected with miR-4721 with/without GSK3β plasmid were determined by PI staining and flow cytometry (n=3 per group). (C) Working model of miR-4721-GSK3β-WNT signaling regulating axis induced by BART22/C-JUN/Sp1, arrow stands for activation or induction, T-shaped arrow stands for suppression.

Table S1.

The sequences used in this study.

Gene			Sequence
miR-4721 mimics		Sense	5'-UGAGGGCUCCAGGUGACGGUGG-3'
		Antisense	5'-ACUCCCGAGGUCCACUGCCACC3'
Negative control		Sense	5' UUUGUACUACACAAAAGUACUG 3'
		Antisense	5' CUGUUCUUUUGUGUUGUUCUUU 3'
C-JUN	1	Sense	5'-GGCACAGCUUAAACAGAAA dTdT-3'
		Antisense	3'-dTdT CCGUGUCGAAUUUGUCUUU-5'
	2	Sense	5'-CGCAGCAGUUGCAAACAUU dTdT-3'
		Antisense	3'-dTdT GCGUCGUCAACGUUUGUAA-5'
GSK3β	1	Sense	5'-AAGAAUCGAGAGCUCCAGAUC dTdT-3'
		Antisense	3'-dTdT UUCUUAGCUCUCGAGGUCUAG-5'
	2	Sense	5'-AAGUAAUCCACCUCUGGCUAC dTdT-3'
		Antisense	3'-dTdT UUCAUUAGGUGGAGACCGAUG-5'
miR-4721 inhibitor			5'-ACUCCCGAGGUCCACUGCCACC3'
Inhibitor negative control			5'-CUGUUCUUUUGUGUUGUUCUUU-3'
Si-C-JUN-1			5'-GGCACAGCTTAAACAGAAA-3'
Si-C-JUN-2			5'-CGCAGCAGTTGCAAACATT-3'
Si-MAP2K4-1			5'-GGACAGAAGTGGAAATATT-3'
Si-MAP2K4-2			5'-GTATAAAGAGCTTCTGAAA-3'

Table S2.

The primers used in this study.

Primers name		Sequence (5’-3’)
GSK3β	Forward	GTCCGATTGCGTTATTTC
	Reverse	AAGAGGTTCTGCGGTTTA
GAPDH	Forward	GGAGCGAGATCCCTCCAAAAT
	Reverse	GGCTGTTGTCATACTTCTCATGG
U6	Forward	CTCGCTTCGGCAGCACA
	Reverse	AACGCTTCACGAATTTGCGT
miR-4721		UGAGGGCUCCAGGUGACGGUGG
Promoter of miR-4721-A	Forward	GAAGAGTTAGAGATTGGAGGAGGTT
	Reverse	CAGTGCTAGAGGCAGAGCTTAGA
Promoter of miR-4721-B	Forward	ATCACCATCAATGCGGCTCAT
	Reverse	CAGTCTGTGTGGGCGTAGTG
Promoter of miR-4721-C	Forward	CTCTGCTCTCTGTGCCCTTGA
	Reverse	GCCACTTCCCAGACACAAAGC

Table S3.

A list of antibodies used for WB, ChIP, EMSA, Co-IP, IF and IHC.

Antibodies name	Cat. No	Company	Species	Dilution
Ki67	9449	CST	Mouse	1:400 (IHC)
β-catenin	8480	CST	Rabbit	1:1000 (WB); 1:100 (IF)
PCNA	13110	CST	Rabbit	1:8000 (IHC)
P21	2947	CST	Rabbit	1:1000 (WB)
SP1	9389	CST	Rabbit	1:1000 (WB)
IgG	2729	CST	Rabbit	1-5ul(IP)
CCND1	55506	CST	Rabbit	1:1000 (WB); 1:400 (IHC)
β-actin	60008-1-Ig	Proteintech	Mouse	1:5000 (WB)
c-JUN	24909-1-AP	Proteintech	Rabbit	1:1000 (WB)
c-Myc	10828-1-AP	Proteintech	Rabbit	1:1000 (WB); 1:200 (IHC)
GSK3β	22104-1-AP	Proteintech	Rabbit	1:1000 (WB); 1:100 (IHC)
GAPDH	CW0100M	CWbio	Mouse	1:1000 (WB)

Table S4.

The sequences used in Electrophoretic mobility shift assay.

Gene	Type		Sequence (5’-3’)
miR-4721	probes	wild type	GTTCAGGGGCGTGGTCTAAGCTCT
	competitors	wild type	GTTCAGGGGCGTGGTCTAAGCTCT
		mutant	GTTCAATACTACCGTCTAAGCTCT
